# Differentiating Cardiac Troponin Levels During Cardiac Myosin Inhibition or Cardiac Myosin Activation Treatments: Drug Effect or the Canary in the Coal Mine?

**DOI:** 10.1007/s11897-023-00620-2

**Published:** 2023-10-25

**Authors:** Matthew M. Y. Lee, Ahmad Masri

**Affiliations:** 1 British Heart Foundation Glasgow Cardiovascular Research Centre, University of Glasgow, Glasgow, UK; 2 Knight Cardiovascular Institute, Oregon Health & Science University, Portland, OR USA

**Keywords:** Cardiac myosin activator, Cardiac myosin inhibitor, Heart failure, Hypertrophic cardiomyopathy, Biomarkers, Troponin

## Abstract

**Purpose of Review:**

Cardiac myosin inhibitors (CMIs) and activators are emerging therapies for hypertrophic cardiomyopathy (HCM) and heart failure with reduced ejection fraction (HFrEF), respectively. However, their effects on cardiac troponin levels, a biomarker of myocardial injury, are incompletely understood.

**Recent Findings:**

In patients with HCM, CMIs cause substantial reductions in cardiac troponin levels which are reversible after stopping treatment. In patients with HFrEF, cardiac myosin activator (omecamtiv mecarbil) therapy cause modest increases in cardiac troponin levels which are reversible following treatment cessation and not associated with myocardial ischaemia or infarction.

**Summary:**

Transient changes in cardiac troponin levels might reflect alterations in cardiac contractility and mechanical stress. Such transient changes might not indicate cardiac injury and do not appear to be associated with adverse outcomes in the short to intermediate term. Longitudinal changes in troponin levels vary depending on the population and treatment. Further research is needed to elucidate mechanisms underlying changes in troponin levels.

**Supplementary Information:**

The online version contains supplementary material available at 10.1007/s11897-023-00620-2.

## Introduction

Cardiac myosin inhibitors and activators are emerging therapies developed for patients with hypertrophic cardiomyopathy (HCM) and heart failure with reduced ejection fraction (HFrEF), respectively. These treatments can affect cardiac troponin levels, a biomarker that is widely used to detect myocardial injury. The interpretation of troponin level changes during treatments can be challenging, as various factors can affect troponin levels. This article reviews the literature on differentiating cardiac troponin levels during cardiac myosin inhibition or activation treatments.

First, we discuss the biology of cardiac muscle which serves as the foundation for understanding cardiac myosin inhibitor and activator therapies. Second, we discuss the role of troponin assays, including in patients with HCM and HFrEF. Third, we describe cardiac myosin inhibitors in HCM and their effects on troponin levels. Fourth, we describe cardiac myosin activators in HFrEF and their effects on troponin levels. Fifth, we conclude by summarising guidance for interpreting troponin levels, and discuss future research directions.

## Cardiac Muscle Biology

**Fig. 1:**
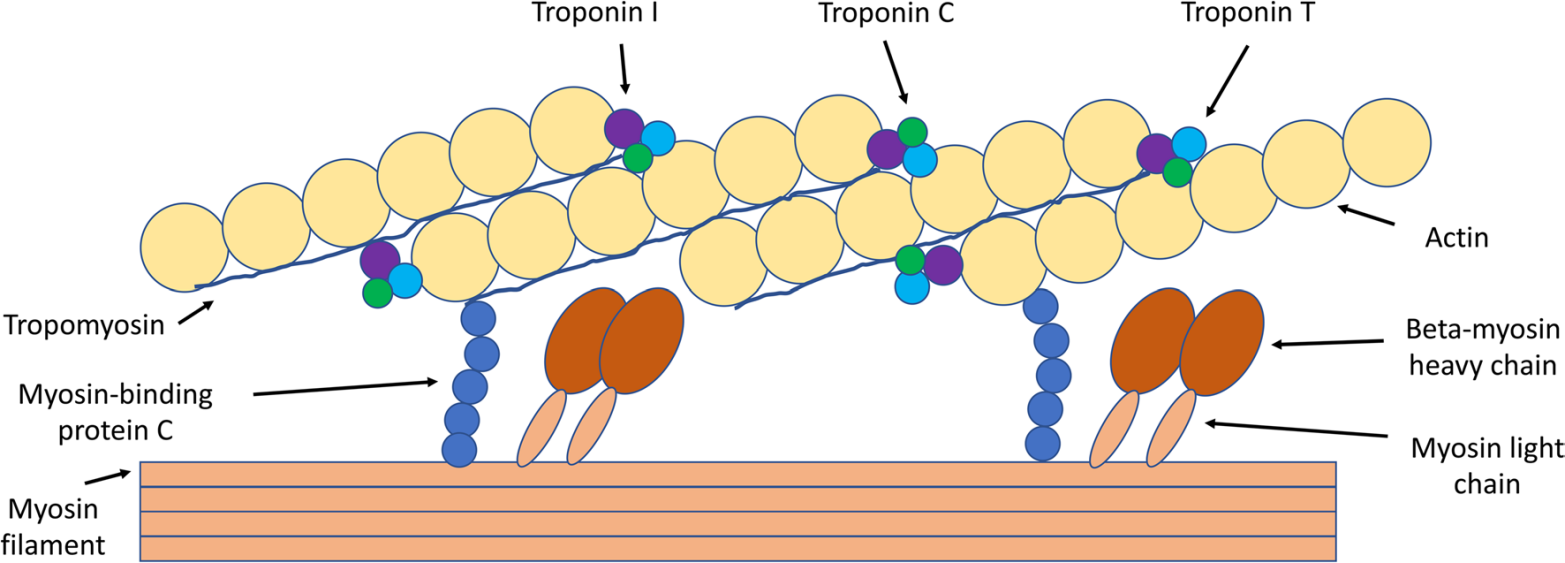
Sarcomere, actin, myosin, and troponin

Striated muscles convert chemical energy to physical work and are comprised of skeletal muscle or cardiac muscle. A sarcomere is the basic contractile unit of cardiac muscle composed of thin actin and thick myosin myofilaments (Fig. 1). Muscle contraction occurs when myosin filaments pull actin filaments closer together, thus shortening the sarcomere unit.

Cardiac myosin is the molecular motor that powers cardiac contraction by converting chemical energy from adenosine triphosphate (ATP) hydrolysis into mechanical force [1]. Cardiac myosin is a hexamer composed of two protein units of β- or α-myosin heavy chain and four myosin light chain molecules [2]. The heavy chains are responsible for ATP hydrolysis and force generation, while the light chains modulate myosin activity. The globular heads of myosin bind actin forming cross-bridges between the thick and thin filaments. The thin actin filament is closely associated with the regulatory troponin complex and α-tropomyosin. Cardiac myosin binding protein-C (MYBPC) contributes to the regulation of contraction.

Cardiac troponin is a protein complex of three subunits (troponin C; the Ca^2+^-binding subunit; troponin I, the inhibitory subunit; and troponin T, the tropomyosin-binding subunit) that regulate the contractility of cardiac muscle [3]. Troponin I and T are cardiac-specific isoforms that are released into the bloodstream after cardiac injury.

## Troponin as a Biomarker

Over the last 2 decades, blood levels of cardiac troponin have transformed clinical care, as the standard biomarker to detect cardiac injury, most frequently used to diagnose or exclude acute coronary syndromes (ACS). Whilst troponin elevation is organ-specific for myocardial injury, it is not disease-specific and clinicians frequently encounter causes of troponin elevation other than ACS such as cardiomyopathies (e.g., HCM), heart failure (HF), tachycardia (e.g., atrial fibrillation), inflammation (e.g., myocarditis), renal dysfunction, anaemia, infection, and medications [4].

Cardiac troponin levels can be useful for diagnosis (ACS, myocarditis), screening (cardiac amyloidosis), prognosis (ACS, HF, HCM), monitor disease progression (myocarditis, HCM), and to monitor treatment efficacy and safety (coronary revascularisation, chemotherapy). For diagnosis, serial testing is recommended to rule out ACS. In patients with pericarditis, increased troponin levels could indicate myocardial involvement (myopericarditis). Elevated troponin levels might justify further evaluation for coronary artery disease, especially if not known prior, and if the pretest probability of coronary artery disease is higher. Elevated troponin levels may prompt clinicians to escalate or favour more intensive therapy during follow-up. A rise in troponin levels post-chemotherapy might indicate cardiotoxicity.

The universal definition of myocardial infarction, from its first version (2007) to its fourth version (2018), provides clinicians guidance on the interpretation of troponin elevations and serial changes (i.e., rise and fall) [Supplemental Table S1] [5•, 6•]. Troponin levels may remain elevated for days or weeks after the onset of cardiac injury, with the rate of declining troponin levels varying depending on disease severity, assay type, time interval measured, treatments received, and patient-specific clearance factors. It is important to consider patient- and laboratory-specific factors when interpretating cardiac troponin levels.

Patient factors such as age- and gender-specific reference ranges for troponin should be considered [7, 8]. Higher body mass index has been associated with increased likelihood of detectable cardiac troponin T (cTnT) levels [9]. A diurnal variation in cTnT has been described [10]. Cardiac troponin levels may be elevated postexercise even in apparently healthy individuals [11]. Troponin is attached to the myocyte contractile apparatus or detached from it in the cytosol—release of cytosolic troponin is proposed to account for the rise in troponin levels with exercise [12, 13].

Cardiac troponin assays vary between manufacturers and should not be used interchangeably. Using a single assay and a central core lab could help decrease variability, especially in multi-centre trials [6•]. A standardised approach is recommended to establish the 99th percentile upper reference limit (URL) for assays. False positive elevations in cardiac troponin can be caused by fibrin clots, heterophilic antibodies, alkaline phosphatase, rheumatoid factor, and cross-reactions of diagnostic (anti-cardiac troponin) antibodies with skeletal troponins [14]. Biotin supplementation may also interfere with troponin assays [15].

In the development of novel therapies, biomarkers such as cardiac troponin may help identify signals of myocardial damage earlier [16]. Avoiding myocardial injury or improving it could be a secondary clinical trial endpoint [17]. In cardio-oncology, elevated troponin pre-therapy is a risk factor for cancer therapy-related cardiomyopathy [18]. An increase in troponin after chemotherapy is a strong predictor of poor cardiac outcome. Cardiac troponin levels might help identify patients with a higher risk profile who are likely to benefit most from specific therapies. In a randomized study of 114 patients with a post-treatment (high-dose chemotherapy) troponin rise, treatment with enalapril seemed to prevent the development of late cardiotoxicity [19].

There are new developments in troponin assays. High sensitivity assays have become routinely available. Transdermal troponin has recently been shown to be clinically feasible for rapid, bloodless prediction of elevated cTnI levels in a study of 238 patients with ACS but clinical application remains a question and requires further investigation [20].

### Interpreting Troponin Levels in HCM and HFrEF

HCM is a genetic heart muscle disease of the cardiac sarcomere. In approximately 40–50% of patients, HCM is caused by mutations in sarcomeric protein genes, most of which are in β-cardiac myosin (MYH7) and cardiac MYBPC [2]. HCM is characterised by myofilament disarray, myocardial hypercontractility, leading to left ventricular hypertrophy (unexplained by loading conditions), and fibrosis [21]. Proposed molecular mechanisms to explain the hypercontractile phenomenon include alterations in the actin-activated β-cardiac myosin chemo-mechanical ATPase cycle, an increased number of functionally accessible myosin heads (i.e., decrease in the super-relaxed state of myosin), and alterations in load dependence contractility that changes the power output of cardiac contraction  [22, 23].

These pathophysiological changes contribute to left ventricular outflow tract (LVOT) obstruction, mitral regurgitation, diastolic dysfunction, myocardial ischaemia, arrhythmias, and autonomic dysfunction. These may cause exertional dyspnoea, fatigue, chest pain, exercise intolerance, palpitations, presyncope/syncope, and sudden cardiac death. Angina in the absence of epicardial coronary artery disease usually occurs with exertion and may result from inability of the coronary microcirculation to supply hypertrophied myocardium, and in obstructive HCM, high myocardial oxygen demand is associated with elevated left ventricular (LV) systolic pressure [24]. Adverse remodelling is defined by the presence of unfavourable structural modifications, translating into increasing LV fibrosis and worsening function, seen in about 15% to 20% of patients with HCM, a smaller proportion of whom will progress to HF [25].

Serum cardiac troponin is elevated in a significant proportion (ranging from 22 to 74%) of patients with HCM and is associated with clinical markers of disease severity including LVOT gradient (LVOT-G), left atrial diameter, LV mass, and fibrosis (as measured by late gadolinium enhancement on cardiovascular magnetic resonance imaging) [Supplemental Table S2]. In patients with HCM, elevated cTnT predicts clinical outcomes i.e., HF (hazard ratio 4.3 for New York Heart Association (NYHA) class II and hazard ratio 22.8 for NYHA class III), atrial fibrillation, and death [26]. Conversely, normal baseline cTnI has a 98% negative predictive value for adverse outcomes [26].

The clinical risk prediction model for sudden cardiac death in HCM (HCM Risk-SCD) recommended by the 2014 European Society of Cardiology (ESC) guidelines for HCM does not incorporate cardiac troponin [27]. However, the addition of cardiac troponin may be useful as an adjunct to current risk models in identifying patients with HCM and adverse cardiac remodelling [28].

To mitigate against postexercise elevations in cardiac troponin, clinical trial protocols generally have prespecified cardiac troponin testing prior to exercise stress echocardiography and avoiding significant activity prior to sample collection [29]. In a study of 127 patients with HCM versus 53 mutation carriers without hypertrophy (controls), patients with HCM were more likely to experience a postexercise increase in troponin compared with mutation carriers (18% vs 4%) [30]. In the HCM group, those who experienced a postexercise troponin increase had higher maximum heart rates and maximal wall thickness, and were more likely to have late gadolinium enhancement on cardiovascular magnetic resonance (CMR). Those with a postexercise increase in troponin were more likely to have high T2 measured on CMR. High T2 was the only independent predictor of troponin rise.

Many patients with HCM have been discouraged from exercising due to concerns about the risk of cardiac events. Several studies have examined the effect of exercise in patients with HCM. The Lifestyle and Exercise in HCM (LIVE-HCM) trial enrolled 1534 individuals with HCM and showed that those who exercised vigorously did not have an increased incidence of serious cardiac events over 3 years of follow-up compared with those who exercised moderately or were inactive [31, 32]. The Randomized Exploratory Study of Exercise Training in HCM (RESET-HCM) trial included 136 patients with HCM, and showed that moderate-intensity exercise compared with usual activity resulted in a small increase in exercise capacity as measured by peak oxygen consumption (pVO_2_) (between-group difference 1.27 [95% CI 0.17–2.37] mL/kg/min; *p* = 0.02) at 16 weeks [33].

Troponin levels are discussed in international guidelines for HCM and HF. In the 2014 ESC guidelines for HCM [34•], laboratory testing for troponin T is recommended as high levels of cTnT are associated with higher risk of cardiovascular events, HF, and death. The 2020 American College of Cardiology (ACC)/American Heart Association (AHA) guidelines for HCM do not provide any specific recommendations on the use of troponin levels [35•].

In the 2021 ESC guidelines for HF, initial laboratory exams recommended include troponin for exclusion of ACS, although elevated levels are detected in the vast majority of patients with AHF [36•]. In patients with suspected myocarditis, troponin is recommended as a mandatory diagnostic test because elevated troponins with dynamic changes are consistent with myocardial necrosis. In HF, persistently elevated troponin levels are a red flag for cardiac amyloidosis. In the 2022 AHA/ACC/Heart Failure Society of America (HFSA) guidelines for HF [18], evidence supporting stage B pre-HF includes patients with risk factors and persistently elevated cardiac troponin in the absence of competing diagnoses resulting in such biomarker elevations such as ACS, chronic kidney disease, pulmonary embolism, or myopericarditis.

## Cardiac Myosin Inhibitors (CMIs) Development

HCM has become a treatable genetic heart disease with low mortality [37]. Traditionally, medical treatment for patients with HCM included beta-blockers, verapamil, diltiazem, and disopyramide as recommended in international guidelines [34•, 35•]. Treatment for HCM has been limited to symptomatic relief without tackling the root cause of the disease, excessive sarcomere contractility. Consequently, there is an unmet need for new therapies that can target the underlying pathophysiology of HCM.

CMIs have been recently developed as a therapy for HCM to directly reduce the myocardial hypercontractility that underlies the pathophysiology of HCM. CMIs target the myofilament apparatus to decrease the number of actin-myosin cross-bridges, thus resulting in dose-dependent reduction in contractility [38, 39]. As CMIs are a targeted disease-specific therapy, they promise less side-effects compared to non-targeted therapy [38, 40].

Mavacamten (formerly MYK-461) is the first-in-class, cardiac-specific, oral small molecule allosteric modulator of β-cardiac myosin that reversibly inhibits its binding to actin [41, 42]. Mavacamten has been approved by the U.S. Food and Drug Administration (FDA) in April 2022 to treat adults with NYHA class II–III obstructive HCM to improve exercise capacity and symptoms [43]. Aficamten (formerly CK-3773274 or CK-274) is a second in-class investigational oral small molecule allosteric inhibitor of cardiac myosin. The favourable pharmacokinetics of aficamten allows for rapid dose adjustments and rapid reversibility after discontinuation [Supplemental Table S3] [38, 41, 44]. Aficamten has minimal drug-drug interactions, with no significant cytochrome P inhibition or induction.

**Fig. 2:**
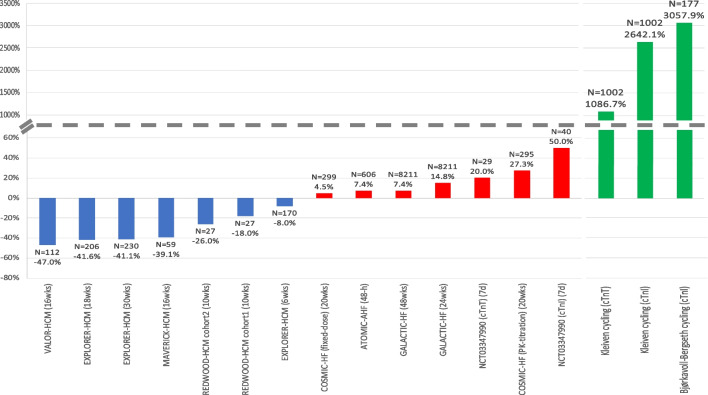
Percentage change from baseline in troponin levels in HCM randomized trials investigating cardiac myosin inhibitors (blue), HFrEF randomized trials investigating cardiac myosin activators (red), and observational studies of exercise (green). N indicates number of patients with data available at follow-up. If between-group difference is not reported: percentage change from baseline in troponin levels = ((change in intervention group—change in control group) / baseline value in intervention group) × 100%. In exercise studies, troponin levels were measured 24-h before and 3-h after 91-km mountain bike race. cTnI, cardiac troponin-I; cTnT, cardiac troponin-T; HCM, hypertrophic cardiomyopathy; HFrEF, heart failure with reduced ejection fraction; wks, weeks

There are reported and ongoing trials investigating CMIs in patients with HCM summarized in Tables 1 and 2, and Fig. 2 and Supplemental Fig. 1A.

**Table 1:**
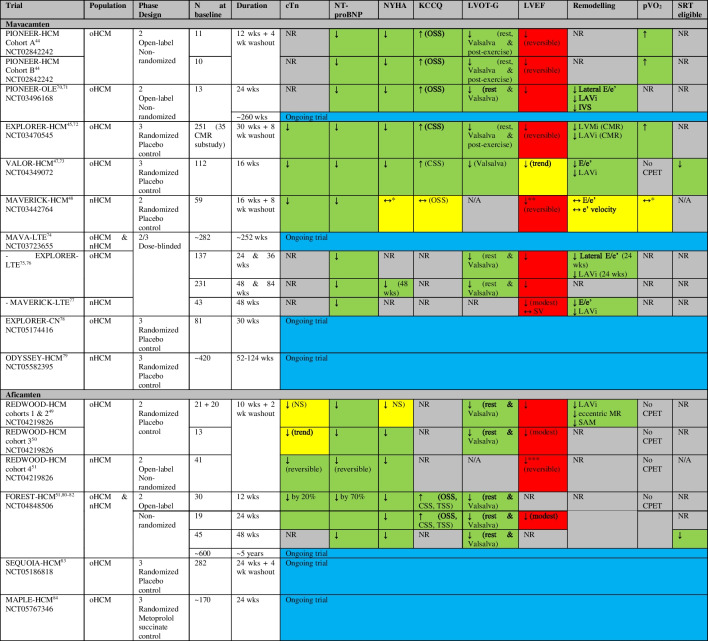
Randomized and non-randomized trials of cardiac myosin inhibitors in HCM and change in troponin levels (and other outcomes)

Green = improvement, yellow = unchanged/not significant, red = deterioration, grey = not reported, blue = ongoing trial

PIONEER-HCM: cohort A (mavacamten dose 10-20 mg/d, without background medications), cohort B (mavacamten dose 2-5 mg/d, with beta-blockers allowed)

EXPLORER-HCM is the only trial that reported the outcome Hypertrophic Cardiomyopathy Symptom Questionnaire (HCMSQ-SoB), which improved with mavacamten therapy, compared to placebo

MAVA-LTE includes patients who have completed EXPLORER-HCM (EXPLORER-LTE cohort) and MAVERICK-HCM (MAVERICK-LTE cohort)

REDWOOD-HCM: cohort 1 (aficamten dose 5–15 mg), cohort 2 (aficamten dose 10–30 mg), cohort 3 (concomitant disopyramide therapy)

FOREST-HCM (CY 6022) (formerly REDWOOD-HCM OLE) is recruiting patients who have completed REDWOOD-HCM or SEQUOIA-HCM

SEQUOIA-HCM (CY 6031)

*MAVERICK-HCM: Composite functional endpoint is defined as either improvement from baseline to week 16 of at least 1.5 mL/kg/min in pVO_2_ and reduction of ≥ 1 in NYHA functional class, or improvement of at least 3.0 mL/kg/min in pVO_2_ and no worsening in NYHA functional class (type II). In subgroup with elevated cTnI (> 99th percentile) or E/e’ average (> 14) at baseline (21 mavacamten, 12 placebo), 33% in mavacamten group met composite functional endpoint vs none in placebo-group (*p* = 0.03)

**MAVERICK-HCM: 5 patients ↓ LVEF ≤ 45% (reversible) (cTnI levels remained less than the 99th percentile in all 5 participants)

***REDWOOD-HCM cohort 4: Three patients (7.3%) had LVEF <50% at week 10; all three patients returned to baseline LVEF after the 2-week washout period. No adverse events of heart failure were reported

*CMR* cardiovascular magnetic resonance, *CPET* cardiopulmonary exercise test, *CSS* clinical summary score, *cTn* cardiac troponin, *IVS* interventricular septal thickness, *KCCQ* Kansas City Cardiomyopathy Questionnaire, *LAVi* left atrial volume indexed, *LVEF* left ventricular ejection fraction, *LVMi* left ventricular mass index, *MR* mitral regurgitation, *nHCM* non-obstructive hypertrophic cardiomyopathy, *NR* not reported, *NS* not significant, *NT-proBNP* N-terminal pro-B-type natriuretic peptide, *NYHA* New York Heart Association, *oHCM* obstructive hypertrophic cardiomyopathy, *OSS* overall summary score, *Ph* phase, *pVO*_2_ peak oxygen consumption, *QoL* quality of life, *SAM* systolic anterior motion, *SRT* septal reduction therapy, *SV* stroke volume, *TSS* total symptom score, *wk* week

**Table 2:**
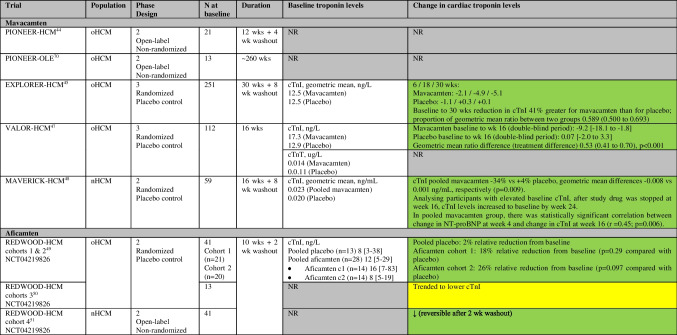
Randomized and non-randomized trials of cardiac myosin inhibitors in HCM and change in troponin levels

Green = improvement, yellow = unchanged/not significant, grey = not reported

*cTnI* cardiac troponin-I, *cTnT* cardiac troponin-T, *nHCM* non-obstructive hypertrophic cardiomyopathy, *NR* not reported, *NT-proBNP* N-terminal pro-B-type natriuretic peptide, *oHCM* obstructive hypertrophic cardiomyopathy, *wk* week

### Mavacamten completed trials

In a non-randomized clinical trial (A Phase 2 Open-label Pilot Study Evaluating MYK-461 in Subjects With Symptomatic HCM and LVOT Obstruction (PIONEER-HCM)) of 21 symptomatic patients with obstructive HCM, mavacamten reduced LVOT obstruction, improved exercise capacity, and symptoms [44]. Subsequently, a randomized, double-blind, placebo-controlled phase 3 trial, Mavacamten for Treatment of Symptomatic Obstructive HCM (EXPLORER-HCM), showed that mavacamten improved exercise capacity, symptoms, and LVOT obstruction in 251 patients with obstructive HCM [45••]. In the EXPLORER-HCM CMR substudy (35 patients), mavacamten treatment resulted in beneficial effects on cardiac remodelling, with reductions in absolute intracellular myocardial mass index, left ventricular mass index, maximum LV wall thickness, and left atrial volume indexed—all predictors of poor prognosis in obstructive HCM [46]. A Study to Evaluate Mavacamten in Adults with Symptomatic Obstructive HCM who are Eligible for Septal Reduction Therapy (VALOR-HCM) was a randomized double-blind, placebo-controlled phase 3 trial that enrolled 112 patients with obstructive HCM with intractable symptoms and showed that mavacamten significantly reduced the fraction of patients meeting guideline criteria for septal reduction therapy after 16 weeks [47••]. In a randomized double-blind, placebo-controlled, phase 2 study (Mavacamten in Adults With Symptomatic Non-Obstructive HCM (MAVERICK-HCM)) which randomized 59 subjects with symptomatic non-obstructive HCM with elevated N-terminal pro-B-type natriuretic peptide (NT-proBNP) ≥ 300 pg/mL, mavacamten was well tolerated and reduced NT-proBNP and cTnI levels but resulted in a reversible decline of LVEF ≤ 45% in 12.5% of patients [48••].

### Mavacamten ongoing trials

 The Extension Study of Mavacamten (MYK-461) in Adults With Symptomatic Obstructive HCM Previously Enrolled in PIONEER (PIONEER-OLE; NCT03496168) and A Long-Term Safety Extension Study of Mavacamten in Adults Who Have Completed MAVERICK-HCM or EXPLORER-HCM (MAVA-LTE; NCT03723655) studies are evaluating the long-term safety of mavacamten. The long-term extension study of VALOR-HCM is ongoing. A Study of Mavacamten in Non-Obstructive HCM (ODYSSEY-HCM; NCT05582395) is a randomized phase III trial designed to investigate the effect of mavacamten versus placebo on Kansas City Cardiomyopathy Questionnaire and pVO_2_.

### Aficamten completed trials

 In a phase I clinical trial in healthy adults, aficamten was well tolerated, adverse events were generally mild and comparable in frequency to those seen with placebo [39]. The double-blind, placebo-controlled, dose-finding Randomized Evaluation of Dosing With CK-274 in Obstructive Outflow Disease in HCM (REDWOOD-HCM) phase 2 trial enrolled a total of 95 patients across 4 cohorts. In cohorts 1 (5–15 mg) and 2 (10–30 mg) which enrolled 41 patients with obstructive HCM and LVOT obstruction, compared with placebo, aficamten reduced LVOT-G, paralleled by improvements in NT-proBNP at 10 weeks [49••]. In cohort 3 which recruited 13 patients with symptomatic obstructive HCM whose background therapy included disopyramide, aficamten treatment substantially reduced LVOT-G and improved NT-proBNP at 10 weeks [50••]. In cohort 4 which recruited 41 patients with non-obstructive HCM, aficamten treatment improved HF symptoms and NT-proBNP at 10 weeks [51••].

### Aficamten ongoing trials

The Safety, Efficacy, and Quantitative Understanding of Obstruction Impact of Aficamten in HCM (SEQUOIA-HCM; NCT05186818) is an ongoing phase 3, randomized, placebo-controlled, double-blind trial assessing the efficacy and safety of aficamten on exercise capacity (pVO_2_ on cardiopulmonary exercise test), HF symptoms, and LVOT-G which has enrolled 282 symptomatic patients with obstructive HCM. The ongoing Follow-up, Open-Label, Research Evaluation of Sustained Treatment with Aficamten in HCM (FOREST-HCM; NCT04848506) trial is evaluating long-term outcomes with aficamten. A Phase 3, Multi-centre, Randomized, Double-blind Trial to Evaluate the Efficacy and Safety of Aficamten Compared to Metoprolol in Adults With Symptomatic Obstructive HCM (MAPLE-HCM; NCT05767346) is a trial with a head-to-head comparison of aficamten with the beta-blocker metoprolol succinate.

Clinical trials in HCM face several challenges. First, event rates of ‘hard endpoints’ e.g., mortality are relatively low, thus very large sample sizes or prolonged follow-up to accrue events would be required to power trials. Therefore, surrogate endpoints, such as how patients ‘feel and function’ endpoints, as well as biomarkers such as NT-proBNP and troponin levels, are used. Second, patients with HCM are heterogeneous and have a wide phenotype (obstructive versus non-obstructive; septal versus apical versus mid-ventricular hypertrophy; genotype positive versus negative; absence/presence of treatment with beta-blockers, calcium-channel blockers, and disopyramide, pre- versus post-septal reduction therapy). Third, CMIs reduce myocardial contractility, which may be clinically evident as a reduction in left ventricular ejection fraction (LVEF). Therefore, clinical trials of CMIs exclude patients with reduced LVEF, and incorporate rigorous echocardiographic monitoring for titration and safety, with down-titration and discontinuation criteria if reduced LVEF occurs. However, reduction in LVEF is in the mechanism of action of CMIs, and currently, it is unclear whether this excessive reversible reduction in LVEF is associated with adverse outcomes.

### Effect of CMIs on Cardiac Troponin Levels

Several mavacamten trials have reported troponin outcomes (Tables 1 and 2) (Fig. 2 and Supplemental Fig. 1A). In EXPLORER-HCM, compared to placebo, mavacamten resulted in a significant and sustained reduction in cTnI levels over 30 weeks, even when LVOT-G did not decrease below commonly used thresholds to define LVOT obstruction [45••, 52]. In MAVERICK-HCM, compared to placebo, mavacamten resulted in rapid sustained improvements in cTnI concentrations over 16 weeks, particularly in those with elevated levels at baseline, despite no significant improvement in pVO_2_ or symptoms [48••]. In MAVERICK-HCM, cTnI results below the limit of detection (0.01 ng/mL) were imputed as one-half the limit (e.g., 0.005 ng/mL) for analysis which is one of the weaknesses of using troponin as an endpoint since many patients do not have elevated troponin level at baseline. Amongst those with elevated baseline cTnI in MAVERICK-HCM, after stopping study drug at week 16, cTnI levels increased to baseline levels by week 24. In the pooled mavacamten group in MAVERICK-HCM, change in NT-proBNP at week 4 correlated with change in cTnI at week 16 (*r* = 0.45, *p* = 0.006). In VALOR-HCM, compared to placebo, mavacamten reduced cTnI levels (geometric mean ratio difference 0.53 (95% CI 0.41 to 0.70), *p* < 0.001) [47••].

One aficamten trial (REDWOOD-HCM) with 4 cohorts has reported troponin levels. In cohorts 1 and 2, aficamten treatment was associated with marked reductions in NT-proBNP, and non-significant reductions in troponin levels (18% relative reduction (*p* = 0.29 compared with placebo) and 26% relative reduction (*p* = 0.097 compared with placebo) in cohorts 1 and 2, respectively). These findings suggest that aficamten may result in other potential downstream pathophysiologic benefits including decreases in LV wall stress and reduction in myocardial injury [49••]. Whether the mechanism of biomarker improvement is primarily related to normalization of LV systolic pressure, improved microvascular blood flow, or other downstream effects of direct myosin modulation requires further study [49••]. In cohort 3, patients on aficamten treatment trended to lower cTnI [50••]. In cohort 4, aficamten treatment reduced cTnI at each study visit up to 10 weeks, and after the 2-week washout period, cTnI levels returned to baseline levels [51••]. Interim analyses from FOREST-HCM also show reductions in cardiac troponin levels at 12 and 24 weeks [51••].

One hypothesis is that in patients with HCM, hypercontractility can result in elevated troponin levels in some patients, and treating the hypercontractility with CMIs can decrease troponin levels. Interpretation of troponin levels in patients with HCM on CMIs is dependent on the clinical presentation. CMIs are expected to lower troponin levels, but patients also typically become more active with improved symptoms on CMIs, where activity and exercise can lead to mild increases in troponin levels as well. Furthermore, HCM and CMIs should not result in a sharp and large increase in troponin levels, so other aetiologies should be investigated. Finally, incorporating LVEF with the troponin level ensures that one does not miss systolic dysfunction on CMIs as part of this troponin level fluctuation.

## Cardiac Myosin Activator Development

Inotropic agents can be classified into cardiac calcitropes (which alter intracellular calcium concentrations), myotropes (which affect the molecular motor and scaffolding), and mitotropes (which influence energetics) [53]. Traditional inotropes (i.e., calcitropes) stimulate contractility via energetically costly augmentation of calcium cycling [54]. Inotropic drugs that acutely improve contractility and cardiac output have had unintended adverse outcomes with increased risk of myocardial ischaemia, ventricular arrhythmias, or death in trials [55–57]. These adverse outcomes are believed to be due to excessively increased cardiac energetic demand due to increased cyclic adenosine monophosphate (AMP) signalling and calcium cycling [58], in line with the hypothesis that the failing heart is energy starved [59].

Newer agents such as myotropes—small-molecule cardiac sarcomere activators that directly increase contractility—directly activate the sarcomere, independent of calcium i.e., without activating cyclic AMP signalling or increasing intracellular calcium cycling [53, 60]. Omecamtiv mecarbil (OM) and danicamtiv are selective cardiac myosin activators, also known as a cardiac myotropes, developed for the potential treatment of patients with HFrEF. As a myotrope, OM has no effect on calcium transients [60]. Cardiac myosin activators represent a novel therapeutic option for HFrEF.

Several trials have assessed OM and outcomes in patients with acute and chronic HFrEF which are summarized in Tables 3 and 4, and Fig. 2 and Supplemental Fig. 1B.

**Table 3:**
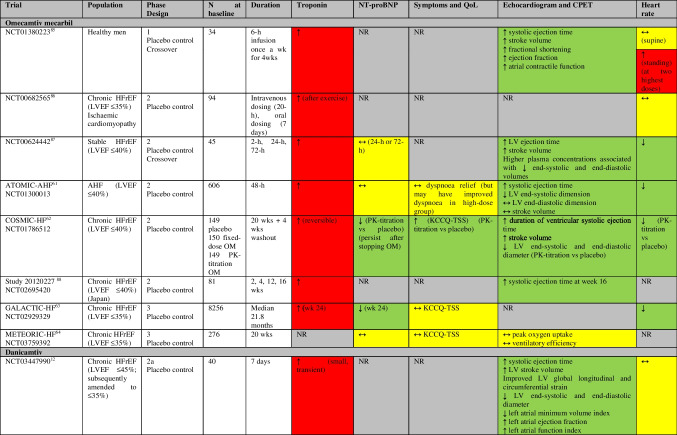
Randomized trials of cardiac myosin activators in HFrEF and change in troponin levels (and other outcomes)

Green = improvement, yellow = unchanged/not significant, red = deterioration, grey = not reported

Please see Table 4 for details of change in troponin levels

*AHF* acute heart failure, *CPET* cardiopulmonary exercise testing, *HFrEF* heart failure with reduced ejection fraction, *KCCQ* Kansas City Cardiomyopathy Questionnaire, *LV* left ventricular, *LVEF* left ventricular ejection fraction, *NR* not reported, *NT-proBNP* N-terminal pro-B-type natriuretic peptide, *OM* omecamtiv mecarbil, *PK* pharmacokinetic, *QoL* quality of life, *TSS* total symptom score, *wk* week

**Table 4:**
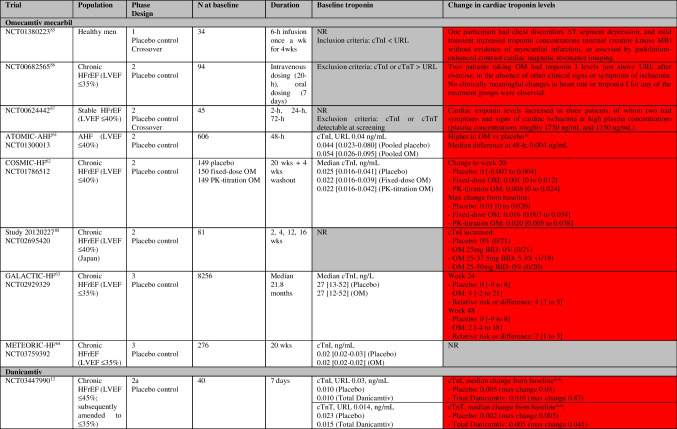
Randomized trials of cardiac myosin activators in healthy volunteers and HFrEF and change in troponin levels

Red = deterioration, grey = not reported

*ATOMIC-AHF: At well tolerated doses (< 1200 ng/mL), small increases in cardiac troponin concentrations were noted in the absence of other clinical evidence of myocardial ischaemia

**NCT03447990: None of the troponin increases observed in the multiple-dose trial were associated with symptoms or with electrocardiogram changes suggestive of ischaemia

*AHF* acute heart failure, *BID* bis in die (two times a day), *cTnI* cardiac troponin-I, *cTnT* cardiac troponin-T, *ECG* electrocardiogram, *HFrEF* heart failure with reduced ejection fraction, *LVEF* left ventricular ejection fraction, *NR* not reported, *NT-proBNP* N-terminal pro-B-type natriuretic peptide, *OM* omecamtiv mecarbil, *PK* pharmacokinetic, *URL* upper reference limit, *wk* week

### Acute HF trials

In the Acute Treatment With Omecamtiv Mecarbil to Increase Contractility in Acute Heart Failure (ATOMIC-AHF) trial enrolling 606 patients admitted for acute HF with LVEF ≤ 40%, a 48-h intravenous infusion of OM treatment did not improve dyspnoea but was generally well tolerated and increased systolic ejection time [61••].

### Chronic HFrEF trials

In a phase 2 trial (Chronic Oral Study of Myosin Activation to Increase Contractility in Heart Failure (COSMIC-HF)) enrolling 448 patients with stable, symptomatic chronic HF and LVEF ≤ 40%, oral OM dosing guided by pharmacokinetics achieved plasma concentrations, improved cardiac function, and decreased ventricular diameter over 20 weeks [62••]. The phase 3 trial (Global Approach to Lowering Adverse Cardiac Outcomes Through Improving Contractility in Heart Failure (GALACTIC-HF)) enrolled 8256 patients with chronic HF and LVEF ≤ 35% and showed that compared to placebo, those who received OM had a lower incidence of a composite of a HF event or cardiovascular death, over a median follow-up of 21.8 months [63••]. In a phase 3 trial (Effect of Omecamtiv Mecarbil on Exercise Capacity in Chronic Heart Failure With Reduced Ejection Fraction (METEORIC-HF)) including 276 patients with chronic HF and LVEF ≤ 35%, compared to placebo, OM did not significantly improve exercise capacity over 20 weeks [64]. In a phase 2a trial enrolling 40 patients with stable HFrEF, danicamtiv was well tolerated and improved LV systolic function and left atrial volume and function [12].

The U.S. FDA issued a briefing document on OM on December 13, 2022, noting that treatment with OM caused a small increase in cardiac biomarkers including cTnI and creatine kinase-MB but acknowledged that the clinical significance of these findings were unclear [65, 66]. However, despite the clear safety of OM in the previously mentioned trials, the small increase in cTnI was debated in the advisory committee meeting as a potential signal of harm. The U.S. FDA issued a complete response letter on February 28, 2023, communicating that GALACTIC-HF alone does not establish substantial evidence of effectiveness sufficient for approval of OM [67].

### Effect of Cardiac Myosin Activators on Cardiac Troponin Levels

Several randomized trials have reported small increases in cardiac troponin levels following treatment with OM (Tables 3 and 4) (Fig. 2 and Supplemental Fig. 1B).

In ATOMIC-AHF, at well tolerated doses (< 1200 ng/mL) of OM, small increases in cTnI concentrations were noted in OM-treated patients compared with placebo (median difference at 48 h, 0.004 ng/mL) in the absence of other clinical evidence of myocardial ischaemia [61••]. However, there was no obvious relationship with OM concentration (*p* = 0.95).

In COSMIC-HF, around a quarter of enrolled patients had cTnI concentrations greater than the 99th percentile URL (0.04 ng/mL) at baseline, with proportions being similar across groups. At week 20, in patients receiving fixed-dose and titrated OM, there was a small increased concentration of circulating cTnI (0.001 ng/mL and 0.006 ng/mL, respectively, compared with no change seen in the placebo group) that did not correlate with the maximum plasma concentration of OM (*r*^2^ = 0.017) [62••]. Possible cardiac ischaemia or infarction were adjudicated by the study’s clinical events committee if investigators reported events suggestive of myocardial ischaemia or if cTnI concentration was > 99th percentile URL of 0·04 ng/mL when the previous concentration had been undetectable, or if the value had increased by > 0·03 ng/mL. Of 278 possible cardiac ischaemia or infarction events associated with increased cTnI concentrations, none of these were deemed to be myocardial infarction following adjudication by the clinical events committee [62••]. Increases in cTnI concentrations returned to baseline values after treatment was stopped.

In GALACTIC-HF, the median cTnI level was higher by 4 and 2 ng/L in the OM group compared to the placebo group, at weeks 24 and 48, respectively [63••]. The incidences of myocardial ischaemia, ventricular arrhythmias, and death were similar in both groups with almost 7500 patient-years of follow-up. Furthermore, no detrimental effects of OM were detected with respect to blood pressure, heart rate, creatinine, or potassium levels.

Although these trials found a small increase in plasma levels of troponin, treatment with OM did not increase the risk of clinical adverse events. The magnitude of troponin release is small in comparison to troponin release in response to exercise in healthy endurance athletes [11] and within the limits of diurnal variation for patients without HF [68]. Excessive exposure to OM may result in prolongation of the systolic ejection time to an extent that theoretically would reduce diastolic coronary blood flow, thus precipitating myocardial ischaemia or infarction [64]. None of the increases in cTnI concentration in the OM program were deemed to indicate myocardial ischaemia, and occurred in the context of improving systolic function, decreasing ventricular volumes, and declining NT-proBNP concentrations. The more likely hypothesis is that further sarcomere recruitment and activation by OM results in the very small increase in troponin observed in OM trials. In comparison, exercise in athletes results in a much higher magnitude in troponin release, although more acute and repetitive rather than chronic elevation, as shown in Fig. 2. Whether other mechanisms are involved, such as exosomal trafficking, requires further investigation [69].

## Conclusion

In patients with both obstructive and non-obstructive HCM, CMIs reduce cardiac troponin levels over short- to medium-term follow-up (10–30 weeks). These reductions in troponin levels are consistent, profound, reversible (after stopping treatment and washout period), and associated with improvements in symptoms, functional capacity, NT-proBNP, and LVOT-G. Long-term data will be critical for characterising the durability of benefit and safety of CMIs to inform potential lifelong therapy in patients with HCM.

In patients with HFrEF, the cardiac myosin activator OM causes a small rise in cardiac troponin levels, which was seen both with acute and chronic treatment, and reversible following treatment cessation. However, these small increases in troponin levels were not associated with increased risk of adjudicated clinical myocardial ischaemia, ventricular arrhythmias, or death, providing reassurance that the biochemical changes do not necessarily equate to adverse clinical outcomes. In February 2023, the U.S. FDA declined the approval of OM for patients with chronic HFrEF [67]. This decision was perhaps, at least in part, swayed by concerns relating to the small and reversible increases in cardiac troponin levels following OM therapy, despite benefits seen in reduction of HF events and cardiovascular death and no evidence of increase in clinical adverse events. This is an example where the use of a biomarker like troponin, if taken out of context, can be detrimental. Exercise leads to significant increase in troponin in the setting of robust sarcomere recruitment that far exceeds what is seen with OM, and appropriately we do not claim that exercise is potentially dangerous due to this rise in troponin.

Troponin levels can be helpful to guide clinicians prior to, during, and after starting cardiac myosin modulators. The predictable rise and fall in cardiac troponin levels might provide an indication of therapeutic efficacy, safety, and importantly, patient adherence with therapy. Troponin levels could also be considered in the future as means to sub-select a population with substantial disease burden. The data accrued in this area are vital given the incredibly high utilization of troponin assays (appropriately and inappropriately), coupled with a likely exponential use of these novel cardiac myosin modulators in routine clinical care.

### Supplementary Information

Below is the link to the electronic supplementary material.

Supplementary file1 (DOCX 143 KB)

## Data Availability

All supporting data are available within the cited references. No new data were generated in support of this review.

## Abbreviations

ACC: American College of Cardiology
ACS: Acute coronary syndrome
AHA: American Heart Association
AMP: Adenosine monophosphate
ATOMIC-AHF: Acute Treatment With Omecamtiv Mecarbil to Increase Contractility in Acute Heart Failure
ATP: Adenosine triphosphate
CMI: Cardiac myosin inhibitor
CMR: Cardiovascular magnetic resonance
COSMIC-HF: Chronic Oral Study of Myosin Activation to Increase Contractility in Heart Failure
cTnI: Cardiac troponin-I
cTnT: Cardiac troponin-T
EMBARK-HFpEF: A Study of Mavacamten in Participants With Heart Failure With Preserved Ejection Fraction and Elevation of NT-proBNP With or Without Elevation of cTnT
ESC: European Society of Cardiology
EXPLORER-HCM: Mavacamten for Treatment of Symptomatic Obstructive HCM
FDA: Food and Drug Administration
FOREST-HCM: Follow-up, Open-Label, Research Evaluation of Sustained Treatment with Aficamten in HCM
GALACTIC-HF: Global Approach to Lowering Adverse Cardiac Outcomes Through Improving Contractility in Heart Failure
HCM: Hypertrophic cardiomyopathy
HF: Heart failure
HFrEF: Heart failure with reduced ejection fraction
HFSA: Heart Failure Society of America
ICD: Implantable cardioverter defibrillator
KCCQ: Kansas City Cardiomyopathy Questionnaire
LIVE-HCM: Lifestyle and Exercise in HCM
LV: Left ventricular
LVEF: Left ventricular ejection fraction
LVOT: Left ventricular outflow tract
LVOT-G: Left ventricular outflow tract gradient
MAPLE-HCM: A Phase 3, Multi-centre, Randomized, Double-blind Trial to Evaluate the Efficacy and Safety of Aficamten Compared to Metoprolol in Adults With Symptomatic Obstructive HCM
MAVA-LTE: A Long-Term Safety Extension Study of Mavacamten in Adults Who Have Completed MAVERICK-HCM or EXPLORER-HCM
MAVERICK-HCM: Mavacamten in Adults With Symptomatic Non-Obstructive HCM
METEORIC-HF: Effect of Omecamtiv Mecarbil on Exercise Capacity in Chronic Heart Failure With Reduced Ejection Fraction
MYBPC: Myosin-binding protein C
NT-proBNP: N-terminal pro-B-type natriuretic peptide
NYHA: New York Heart Association
ODYSSEY-HCM: A Study of Mavacamten in Non-Obstructive HCM
OM: Omecamtiv mecarbil
PIONEER-HCM: A Phase 2 Open-label Pilot Study Evaluating MYK-461 in Subjects With Symptomatic HCM and Left Ventricular Outflow Tract Obstruction
PIONEER-OLE: Extension Study of Mavacamten (MYK-461) in Adults With Symptomatic Obstructive HCM Previously Enrolled in PIONEER
pVO_2_: Peak oxygen consumption
REDWOOD-HCM: Randomized Evaluation of Dosing With CK-274 in Obstructive Outflow Disease in HCM
RESET-HCM: Randomized Exploratory Study of Exercise Training in HCM
SEQUOIA-HCM: Safety, Efficacy, and Quantitative Understanding of Obstruction Impact of Aficamten in HCM
URL: Upper reference limit
VALOR-HCM: A Study to Evaluate Mavacamten in Adults with Symptomatic Obstructive HCM who are Eligible for Septal Reduction Therapy
