# Correction to: Differentiating Cardiac Troponin Levels During Cardiac Myosin Inhibition or Cardiac Myosin Activation Treatments: Drug Effect or the Canary in the Coal Mine?

**DOI:** 10.1007/s11897-023-00639-5

**Published:** 2023-12-01

**Authors:** Matthew M. Y. Lee, Ahmad Masri

**Affiliations:** 1 British Heart Foundation Glasgow Cardiovascular Research Centre, University of Glasgow, Glasgow, UK; 2 Knight Cardiovascular Institute, Oregon Health & Science University, Portland, OR USA

**Correction to** **:** **Curr Heart Fail Rep. 2023**

**https://doi.org/10.1007/s11897-023-00620-2**

1. Figure 2 has been updated with corrected numbers.
2. Online version references 78-88 have been corrected (as per PDF).
3. The title of Table 1, Table 3, and Table 4 had references [87], [88], and [88], respectively. These references were added in error during the proof corrections stage and have now been removed.
4. Added abbreviation PK pharmacokinetics to Table 3 footnote.
5. Removed OM in title of Table 4.

The original version has been corrected.

