# Supplementary material for: Differentiating Cardiac Troponin Levels During Cardiac Myosin Inhibition or Cardiac Myosin Activation Treatments: Drug Effect or the Canary in the Coal Mine?
Source: Curr Heart Fail Rep. 2023 Oct 25;20(6):504–18. doi: 10.1007/s11897-023-00620-2 (PMC10746589; doi:10.1007/s11897-023-00620-2)
Supplement: Supplementary file 1 — Supplementary file1 (DOCX 143 KB) [file 11897_2023_620_MOESM1_ESM.docx]

ONLINE SUPPLEMENTARY MATERIAL

# Lee MMY, Masri A. Differentiating Cardiac Troponin Levels During Cardiac Myosin Inhibition or Cardiac Myosin Activation Treatments: Drug Effect or the Canary in the Coal Mine? Current Heart Failure Reports. 2023. <https://doi.org/10.1007/s11897-023-00620-2>.

# Supplemental Figure 1: Percentage change from baseline in troponin levels in HCM randomized trials investigating cardiac myosin inhibitors (panel A) and HFrEF randomized trials investigating cardiac myosin activators (panel B)

Size indicates number of patients with data available at follow-up. If between-group difference is not reported, percentage change from baseline in troponin levels = ((change in intervention group - change in control group) / baseline value in intervention group) x 100%

cTnI, cardiac troponin-I; cTnT, cardiac troponin-T; HCM, hypertrophic cardiomyopathy; HFrEF, heart failure with reduced ejection fraction; wks, weeks

# Supplemental Table S1. Fourth universal definition of myocardial infarction (2018)

| **Type** | **Criteria** |
| --- | --- |
| 1 | Detection of a rise and/or fall of cTn values with at least one value above the 99^th^ percentile URL and with at least one of the following:   - Symptoms of acute myocardial ischaemia - New ischaemic ECG changes - Development of pathological Q waves - Imaging evidence of new loss of viable myocardium or new regional wall motion abnormality in a pattern consistent with an ischaemic aetiology - Identification of a coronary thrombus by angiography including intracoronary imaging or by autopsy* |
| 2 | Detection of a rise and/or fall of cTn values with at least one value above the 99^th^ percentile URL, and evidence of an imbalance between myocardial oxygen supply and demand unrelated to acute coronary athero-thrombosis, requiring at least one of the following:   - Symptoms of acute myocardial ischaemia - New ischaemic ECG changes - Development of pathological Q waves - Imaging evidence of new loss of viable myocardium or new regional wall motion abnormality in a pattern consistent with an ischaemic aetiology |
| 3 | Patients who suffer cardiac death, with symptoms suggestive of myocardial ischaemia accompanied by presumed new ischaemic ECG changes or ventricular fibrillation, but die before blood samples for biomarkers can be obtained, or before increases in cardiac biomarkers can be identified, or MI is detected by autopsy examination |
| 4a | Myocardial infarction associated with percutaneous coronary intervention.  Coronary intervention-related MI is arbitrarily defined by an elevation of cTn values more than five times the 99^th^ percentile URL in patients with normal baseline values. In patients with elevated pre-procedure cTn in whom the cTn level are stable (≤ 20% variation) or falling, the post-procedure cTn must rise by >20%. However, the absolute post-procedural value must still be at least five times the 99^th^ percentile URL. In addition, one of the following elements is required:   - New ischaemic ECG changes - Development of new pathological Q waves** - Imaging evidence of new loss of viable myocardium or new regional wall motion abnormality in a pattern consistent with an ischaemic aetiology - Angiographic findings consistent with a procedural flow-limiting complication such as coronary dissection, occlusion of a major epicardial artery or a side branch occlusion/thrombus, disruption of collateral flow, or distal embolization*** |
| 4b | Stent/scaffold thrombosis associated with percutaneous coronary intervention |
| 4c | Restenosis associated with percutaneous coronary intervention |
| 5 | Myocardial infarction associated with coronary artery bypass grafting.  CABG-related MI is arbitrarily defined as elevation of cTn values >10 times the 99^th^ percentile URL in patients with normal baseline cTn values. In patients with elevated pre-procedure cTn in whom cTn levels are stable (≤ 20% variation) or falling, the post-procedure cTn must rise by >20%. However, the absolute post-procedural value still must be >10 times the 99^th^ percentile URL. In addition, one of the following elements is required:   - Development of new pathological Q waves**** - Angiographic documented new graft occlusion or new native coronary artery occlusion - Imaging evidence of new loss of viable myocardium or new regional wall motion abnormality in a pattern consistent with an ischaemic aetiology |

*Post-mortem demonstration of an atherothrombus in the artery supplying the infarcted myocardium, or a macroscopically large circumscribed area of necrosis with or without intramyocardial haemorrhage, meets the type 1 MI criteria regardless of cTn values

**Isolated development of new pathological Q waves meets the type 4a MI criteria if cTn values are elevate and rising but less than five times the 99^th^ percentile URL

***Post-mortem demonstration of a procedure-related thrombus in the culprit artery, or a macroscopically large circumscribed area of necrosis with or without intra-myocardial haemorrhage meets the type 4a MI criteria

****Isolated development of new pathological Q waves meets the type 5 MI criteria if cTn values are elevated and rising but <10 times the 99^th^ percentile URL

CABG, coronary artery bypass graft; cTn, cardiac troponin; ECG, electrocardiogram; MI, myocardial infarction; URL, upper reference limit

# Supplemental Table S2. Associations with elevated troponin levels in patients with HCM in observational studies

| **Country, first author, year** | **Size** | **Population** | **Prevalence of elevated troponin** | **Associations with elevated troponin** |
| --- | --- | --- | --- | --- |
| Scotland, Osmanska^1^, 2020 | 313  (204 CMR subgroup) | Adults with established diagnosis of HCM referred to single centre for genetic testing  Median age 57y  64% male  30% pathogenic variant in a sarcomere gene | 69 (22%)  cTnI | 32% female vs 17% male  ↑ LV wall thickness (TTE)  ↑ LVOT-G (TTE)  ↑ LVM (CMR)  ↑ fibrosis (% LGE) (CMR) |
| Japan, Kubo^2^, 2013 | 183 | Consecutive patients with HCM who had clinical evaluation including cTnI  Mean age 61y  62% male  14% LVOT-G ≥30mmHg (rest)  Mean MWT 20.1mm | 99 (54%)  cTnT | 4.1y mean follow-up: ↑ CV events (CV deaths, unplanned HF admissions, sustained VT, embolic events, progression to NYHA III-IV) |
| Netherlands, Gommans^3^, 2021 | 135 | National multicentre cohort of patients with HCM who had clinical evaluation, CMR and biomarker assessment  Mean age 54y  59% male  59% pathogenic mutation  15% LVOT-G ≥30mmHg (rest)  Median MWT 17mm (CMR)  62% LGE presence  27% high T2 | 33 (24%)  cTnT | 5.0y median follow-up: ↑ risk of composite of SCD, HF related death, stroke-related death, HF hospitalization, hospitalization for stroke, spontaneous sustained VT or appropriate ICD discharge, and progression to NYHA class III-IV |
| Scotland, Connelly^4^, 2016 | 100  (49 CMR subgroup) | Consecutive patients with HCM referred to West of Scotland Inherited Cardiac Conditions Clinic  Mean age 56y  60% male  20% LVOT-G ≥30mmHg (rest) | 27 (27%)  cTnI | ↑ HCM-risk score  ↑ LA diameter, ↑ max LVOT-G  ↑ history of AF  ↑ history of HF  ↑ LGE (CMR) |
| Spain, Moreno^5^, 2010 | 95 | Haemodynamically stable HCM  Mean age 46y  76% male | 40 (42%)  cTnT | NYHA ≥3  Outflow obstruction, systolic dysfunction, abnormal blood pressure response  LGE (CMR)  ↑ MWT, ↑ LA diameter, ↑ LVOT-G |
| Netherlands, Gommans^6^, 2013 | 62 | Clinical HCM  Mean age 51y  58% male | 46 (74%)  cTnT | ↑ LVM/BSA (CMR)  ↑ MWT (CMR)  ↑ Fibrosis extent in LGE positive |
| Japan, Kawasaki^7^, 2013 | 53 | Consecutive patients with HCM referred for CMR  Mean age 62y  72% male | Not reported  cTnT | cTnT ≥0.007 ng/mL for detecting LGE PPV 71%, NPV 79%  ↑ LGE extent was related to cTnT level in 8 patients during 22 months of follow-up |
| Germany, Lehrke^8^, 2012 | 52 | Patients with HCM who had CMR | Not reported  cTnT | ↑ T2 hyper-intensities |
| China, Chen^9^, 2020 | 44 | Consecutive patients with HCM referred to hospital and had CMR  Mean age 51y  56% male  41% LVOT-G ≥30mmHg (rest) | 28 (64%)  cTnT | ↑ myocardial high T2 signal (no. of segments and % of myocardium) |

AF, atrial fibrillation; BSA, body surface area; CMR, cardiovascular magnetic resonance; cTnI, cardiac troponin I; cTnT, cardiac troponin T; CV, cardiovascular; HCM, hypertrophic cardiomyopathy; HF, heart failure; ICD, implantable cardioverter defibrillator; LA, left atrium; LGE, late gadolinium enhancement; LV, left ventricular; LVM, left ventricular mass; LVOT-G, left ventricular outflow tract gradient; MWT, maximal wall thickness; NPV, negative predictive value; NYHA, New York Heart Association; PPV, positive predictive value; SCD, sudden cardiac death; TTE, transthoracic echocardiography; VT, ventricular tachycardia

# Supplemental Table S3. Comparison of Mavacamten and Aficamten

| **Pharmacokinetic characteristics** | **Mavacamten (MYK-461)** | **Aficamten (CK-274)** |
| --- | --- | --- |
| Plasma half-life | 7-9 days | 3.4 days |
| Time to achieve steady state | 6 weeks | 2 weeks |
| Therapeutic window | Narrower | Wider |
| Cytochrome P450 induction/inhibition | Higher probability | Lower probability |

# References

1 Osmanska J, Connelly A, Nordin S, et al. High sensitivity troponin I in hypertrophic cardiomyopathy. Eur Heart J. 2020;41(2):ehaa946.2078. <https://doi.org/10.1093/ehjci/ehaa946.2078>.

2 Kubo T, Kitaoka H, Yamanaka S, et al. Significance of high-sensitivity cardiac troponin T in hypertrophic cardiomyopathy. J Am Coll Cardiol. 2013;62(14):1252–9. <https://doi.org/10.1016/j.jacc.2013.03.055>.

3 Gommans DHF, Cramer GE, Fouraux MA, et al. Usefulness of High-Sensitivity Cardiac Troponin T to Predict Long-Term Outcome in Patients with Hypertrophic Cardiomyopathy. Am J Cardiol. 2021;152:120–4. <https://doi.org/10.1016/j.amjcard.2021.04.040>.

4 Connelly A, Coats C, Hunter A, Murday V, Findlay I. 147 Elevated Serum Troponin I Is Associated with Increased Risk in HCM. Heart. 2016;102:A106. <https://doi.org/10.1136/heartjnl-2016-309890.147>.

5 Moreno V, Hernández-Romero D, Vilchez JA, et al. Serum levels of high-sensitivity troponin T: a novel marker for cardiac remodeling in hypertrophic cardiomyopathy. J Card Fail. 2010;16(12):950–6. <https://doi.org/10.1016/j.cardfail.2010.07.245>.

6 Gommans F, Bakker J, Cramer E, et al. Elevated high-sensitivity cardiac troponin is associated with hypertrophy and fibrosis assessed with CMR in patients with hypertrophic cardiomyopathy. J Cardiovasc Magn Reson. 2013;15(Suppl 1):P144. <https://doi.org/10.1186/1532-429X-15-S1-P144>.

7 Kawasaki T, Sakai C, Harimoto K, Yamano M, Miki S, Kamitani T. Usefulness of high-sensitivity cardiac troponin T and brain natriuretic peptide as biomarkers of myocardial fibrosis in patients with hypertrophic cardiomyopathy. Am J Cardiol. 2013;112(6):867–72. <https://doi.org/10.1016/j.amjcard.2013.04.060>.

8 Lehrke S, Lossnitzer D, Viertler D, Giannitsis E, Steen H. T2 abnormalities in patients with hypertrophic cardiomyopathy characterized by cardiovascular magnetic resonance imaging- an indicator of myocardial injury as assessed by the high sensitive cardiac troponin T assay. J Cardiovasc Magn Reson. 2012;14(Suppl 1):O100. <https://doi.org/10.1186/1532-429X-14-S1-O100>.

9 Chen S, Huang L, Zhang Q, Wang J, Chen Y. T2-weighted cardiac magnetic resonance image and myocardial biomarker in hypertrophic cardiomyopathy. Medicine. 2020;99(23):e20134. <https://doi.org/10.1097/MD.0000000000020134>.
